# Transcriptomics for child and adolescent tuberculosis*

**DOI:** 10.1111/imr.13116

**Published:** 2022-07-12

**Authors:** Myrsini Kaforou, Claire Broderick, Ortensia Vito, Michael Levin, Thomas J. Scriba, James A. Seddon

**Affiliations:** ^1^ Department of Infectious Disease Imperial College London London UK; ^2^ South African Tuberculosis Vaccine Initiative, Institute of Infectious Disease and Molecular Medicine and Division of Immunology, Department of Pathology University of Cape Town Cape Town South Africa; ^3^ Desmond Tutu TB Centre, Department of Paediatrics and Child Health Stellenbosch University Cape Town South Africa

**Keywords:** children, diagnosis, differential expression, transcriptomics, tuberculosis

## Abstract

Tuberculosis (TB) in humans is caused by *Mycobacterium tuberculosis* (*Mtb*). It is estimated that 70 million children (<15 years) are currently infected with *Mtb*, with 1.2 million each year progressing to disease. Of these, a quarter die. The risk of progression from *Mtb* infection to disease and from disease to death is dependent on multiple pathogen and host factors. Age is a central component in all these transitions. The natural history of TB in children and adolescents is different to adults, leading to unique challenges in the development of diagnostics, therapeutics, and vaccines. The quantification of RNA transcripts in specific cells or in the peripheral blood, using high‐throughput methods, such as microarray analysis or RNA‐Sequencing, can shed light into the host immune response to *Mtb* during infection and disease, as well as understanding treatment response, disease severity, and vaccination, in a global hypothesis‐free manner. Additionally, gene expression profiling can be used for biomarker discovery, to diagnose disease, predict future disease progression and to monitor response to treatment. Here, we review the role of transcriptomics in children and adolescents, focused mainly on work done in blood, to understand disease biology, and to discriminate disease states to assist clinical decision‐making. In recent years, studies with a specific pediatric and adolescent focus have identified blood gene expression markers with diagnostic or prognostic potential that meet or exceed the current sensitivity and specificity targets for diagnostic tools. Diagnostic and prognostic gene expression signatures identified through high‐throughput methods are currently being translated into diagnostic tests.

## 1 INTRODUCTION

Tuberculosis (TB) is a disease caused by *Mycobacterium tuberculosis* (*Mtb*) and the organism has evolved with humans for >10 000 years.^1^ Individuals with infectious TB generate droplet nuclei containing *Mtb* which, when coughed or breathed out, can remain in the air for several hours. Following inhalation of infected droplets, the bacilli need to overcome physical barriers and antimicrobial peptides, to reach the terminal alveoli, where they encounter elements of the innate immune system, most commonly macrophages and dendritic cells.^2^ If *Mtb* survives this encounter with the innate system and sensitizes the adaptive immune system, as measured by tuberculin skin testing (TST) or interferon gamma release assays (IGRAs), the individual is said to have *Mtb* infection (sometimes termed TB infection or latent TB infection). Commonly, the mycobacteria are contained by the immune system with only low numbers of organisms persisting. However, if the bacilli overcome these constraints and multiply, symptoms and signs of TB disease develop, accompanied by radiological changes in the lungs or other sites of disease. It may be possible to isolate mycobacteria from respiratory samples or samples taken from other sites of disease that can be cultured or identified using molecular tests. Overall, about 10% of individuals with *Mtb* infection will progress to TB disease. Drug therapy given to individuals with *Mtb* infection is effective at preventing this progression and is termed TB preventive therapy (TPT or latent TB treatment). If an individual develops TB disease, then TB disease treatment can be given, which is again successful in most patients.

The natural history of TB in children and adolescents is different to adults. The risk of *Mtb* infection rises with age in a relatively linear way dependent on the prevailing TB prevalence in that context, reflecting cumulative exposure. However, the risk of progressing from *Mtb* infection to TB disease varies with age.^3^ Young children (<5 years of age) are at high risk of disease progression, with the risk falling to a nadir in primary school age children.^4^ This risk rises as children enter puberty, increasing earlier in females but with males then following and eventually overtaking females in adulthood. Work done in the era prior to antibiotics suggests that there is a “timetable” for TB in children, with almost all children who progress to disease doing so within a year or two of infection. In addition, the type of disease seen in children is different to adults. Young children typically have paucibacillary disease, a term implying that few or no organisms are commonly found in respiratory samples that undergo microbiological evaluation. TB disease in this age group usually presents as either intrathoracic lymph node disease or disseminated disease, including TB meningitis or miliary TB.^5^ As children enter adolescence, they begin to develop adult‐type disease with extensive parenchymal involvement and cavities. Large numbers of organisms are commonly isolated from respiratory samples that undergo microbiological evaluation.

The World Health Organization (WHO) defines children as <15 years and adolescents as 10 to <20 years. It is estimated that currently 70 million children have *Mtb* infection and each year 1.2 million develop TB disease.^6^ It is estimated that an additional 535 000 15 to <20‐year‐old develop disease each year.^7^ Only about half of the estimated number of incident cases of child TB each year are diagnosed and treated, and WHO suggests that 230 000 children die of TB annually,^6^ with 96% of these being undiagnosed.^8^ For many years, global and most national guidance has been that children <5 years and those living with HIV who have been exposed to an infectious case of TB should receive TPT. Guidance has been to treat following exposure given that tests of *Mtb* infection are rarely available in high TB‐burden, low‐resource settings. However, of the 1.27 million children estimated to be eligible for TPT in 2017, only 23% received treatment.^9^ The most recent WHO guidance expands TPT provision to also say that all household contacts of infectious TB patients can be given TPT following exclusion of TB disease.^10^ Very few of these individuals receive TPT each year. The WHO End TB Strategy seeks to reduce TB deaths by 95% by 2035, compared with 2015 levels, as well as reduce incidence by 90% over the same period, with children and adolescent comprehensively included in the Strategy. However, it is recognized that using current approaches, global progress will fall far short of these targets.^11^The COVID‐19 pandemic has severely disrupted health services and TB programs. This disruption is almost certainly a factor behind the observed 25–50% fall in the detection and treatment of new TB cases which was observed in just a 3‐month period in 2020.^6^ Though the impact of COVID‐19 control measures on *Mtb* transmission has not yet been defined, it is predicted that reduced case‐finding and treatment during the pandemic will lead to increased TB mortality.^12^, ^13^, ^14^, ^15^

The End TB Strategy suggests that to achieve their ambitious targets new tools will be required. These include point‐of‐care diagnostic tests for TB disease, new tests to identify which individuals will progress to disease in the future, as well as new vaccines and new drugs. To develop these tools, it is increasingly recognized that a more complete understanding of the immune response to *Mtb* is required, as well as a better understanding of how to use host responses to discriminate between clinical groups. One area of host response biology that has evolved substantially over the last 10 years is transcriptomics or the study of RNA expression. In this article, we will discuss the role of transcriptomics in child and adolescent TB, with a particular focus on transcriptomics in blood. We review the literature on studies that have employed transcriptomics to both better understand child and adolescent TB as well as develop diagnostic tests.

## 2 CHALLENGES IN CHILD AND ADOLESCENT TB

**FIGURE 1:**
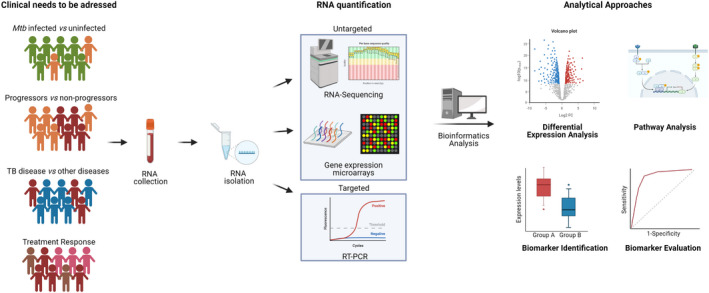
Overview of the role of transcriptomics in pediatric and adolescent TB, together with steps required for RNA quantification and bioinformatics analysis. Created with BioRender.com

There are multiple challenges facing the field of childhood TB, many of which could benefit from the application of transcriptomic approaches (Figure 1). Some of these challenges relate to decision‐making around the clinical management of children, some to the development of new therapies or vaccines, and some to the way that clinical research in child TB is conducted. Below we first outline these challenges and then we systematically describe studies that have been done in this field.

### 2.1 Discriminating children with TB from children with other diseases

Children with TB disease generally have non‐specific symptoms and signs that overlap with other common conditions seen in childhood. Radiological investigations, the most used being chest X‐ray, are also frequently non‐specific with abnormalities that could be consistent with TB but also with other conditions. While TB disease in adults is usually diagnosed through the identification of *Mtb* in the sputum, this type of diagnostic confirmation is uncommon in children. There are two reasons for this. The first is that samples for evaluation can be challenging to obtain. As young children are unable to spontaneously expectorate sputum, more invasive sampling is required, including induced sputum (requiring nebulized hypertonic saline to stimulate coughing followed by aspiration of sputum from the pharynx through the nose), gastric aspiration (the suctioning of stomach contents using a nasogastric tube to collect sputum coughed and swallowed during sleeping), and the collection of stool samples (to identify swallowed *Mtb* that has passed through the digestive system). The second is that even if good quality respiratory samples are collected, microbiological evaluation using culture or molecular diagnostic testing only identifies *Mtb* in a relatively low proportion of children determined clinically to have TB disease (commonly about 20%).^16^ It is assumed that this is due to the presence of few organisms in respiratory samples. Given these challenges, new diagnostic tests are required, ones that do not rely on the microbiological evaluation of respiratory samples.

Of the children who present to health facilities for evaluation of their clinical symptoms and signs, some will have TB, but many will not. Established symptoms required to classify a child as a presumptive TB case include any of the following^17^, ^18^: (a) cough ≥2 weeks, (b) persistent, unexplained lethargy, (c) unexplained fever ≥1 week, (d) poor growth/weight loss over the preceding 3 months, or (e) cough <1 week with a known TB exposure in the previous 12 months. A positive TST or chest X‐ray suggestive of TB are also criteria. A test that can discriminate presumptive TB cases who have TB from presumptive TB cases who have other causes for their symptoms would make a substantial impact on the vast under‐diagnosis of child TB that is seen on a global scale. In turn, this could impact dramatically on child TB mortality.

In addition to children being brought to healthcare services for evaluation (termed passive case finding), active case finding strategies seek to identify new, undiagnosed cases amongst high‐risk populations. Children with HIV, those with malnutrition and those with any degree of immunosuppression should be considered at high risk. It is recommended that these children are regularly screened for TB disease. A further high‐risk group is children recently exposed to infectious cases of TB, given the high proportion with infection and the substantial risk of disease progression in young children with recent exposure. Global guidance and almost all national guidelines suggest that after a new infectious adult TB case is diagnosed, the house should be visited, and all household members evaluated for TB disease. Systematic reviews of the yield of household contact tracing suggest that between 5 and 10% of children screened at home visits have prevalent TB disease.^19^, ^20^ Deciding which children have TB disease, which have other diseases that are causing symptoms, and which are well, can be challenging as again, symptoms, signs, and radiology are non‐specific. A test that could assist in identifying those children who need TB disease treatment would be very beneficial.

### 2.2 Predicting disease progression in TB‐exposed children and adolescents

Following exposure to an infectious case of TB, and following exclusion of TB disease, TPT is advised for young children and children living with HIV.^10^ However, even though these children are at increased risk of developing TB compared with HIV‐negative older children and adults, most of these “high‐risk” children will not progress to TB disease. The only tests that are currently available that assist in decision‐making are the tests of *Mtb* infection, namely the TST and IGRA. These tests signify immunological sensitization by detecting *Mtb*‐specific T‐cell responses, and do not indicate if there are either viable bacilli in the child or if there is a high risk of future disease progression. TB‐exposed children with a positive TST or IGRA are at higher risk of disease progression than children with negative tests but still only a small proportion of those with positive tests will progress to disease.^21^ Children under 5 years with a positive IGRA/TST have a 2‐year incidence of ~20% while in children over 5 years this risk is ~10%. Overall, for all TB‐exposed children (irrespective of infection status), the risk is substantially below 10%. Increasingly there is recognition that older HIV‐negative children and adults should also be given TPT following household exposure.^10^ For these individuals, the risk of future disease progression is even lower.

This means that many well children, adolescents, and adults need to be given treatment to prevent each TB case. TPT involves several months of medication which can be challenging for children and families and puts additional strain on health systems. TPT is also seen as a low priority by health services and families and treatment completion rates for children are very low.^22^ Although rare, adverse events do occur, there are non‐specific effects on the microbiome, and any unnecessary pill burden is ideally avoided. A biomarker that could identify which TB‐exposed children and adolescents are at high risk of disease progression would allow targeting of interventions to those most needing them.

Increasingly the concept of *Mtb* infection and TB disease being dichotomous disease states is being challenged and a dynamic continuum recognised.^23^ Under the most commonly accepted model of this continuum,^24^ those with incipient TB have detectable metabolic activity of *Mtb*, but without any clinical symptoms or signs, radiological abnormalities or positive microbiology for *Mtb*. Those with sub‐clinical disease do not have clinical symptoms or signs but may have radiological changes or it may be possible to isolate *Mtb* from respiratory samples. Individuals with incipient or sub‐clinical disease are more likely to progress to clinically apparent disease and so any biomarker that could identify these clinical states, allowing appropriate treatment, would be valuable.

### 2.3 Identifying children and adolescents who are not responding to TB disease treatment

Treatment outcomes for children diagnosed and treated for TB are generally good with low rates of mortality.^25^ However, there are several groups of children in whom the proportion with unfavorable outcome is higher. These include children with certain forms of TB, most notably TB meningitis,^26^ and children with other conditions complicating their TB treatment, such as HIV co‐infection or malnutrition. Also, if a child is treated for drug‐susceptible TB, based on clinical criteria, while having drug‐resistant TB, then treatment is unlikely to be effective. Children with TB who are not given their TB drugs, or who do not take their drugs, do not do well and if a child is diagnosed with TB based on clinical criteria and fails to respond, one potential reason is that they do not have TB but have another cause for their symptoms, signs, and radiology. In contrast to younger children, treatment outcomes for adolescents are less good,^27^ with high rates of treatment failure and death. In all these instances, it would be useful identify that a child or adolescent is not responding to treatment as early as possible to allow appropriate interventions. In clinical practice, it often takes several months to identify that a child or adolescent is not responding well to treatment. Failure to put on weight, persistence or worsening of symptoms, and worsening radiology all indicate treatment failure, but these take time to detect and are not specific. For adult TB, where most patients are microbiologically confirmed at baseline, sputum smear, or culture conversion at 2 months is used as a surrogate marker.^28^ While this marker is associated with favorable outcome, it is not a sensitive or specific indicator and 2 months is late to be identifying a patient failing therapy. Although this 2‐month microbiological conversion can be used for individuals who were microbiologically confirmed at baseline, this represents a small proportion of children treated for TB.

If it were possible early in therapy to identify children and adolescents who were not responding to treatment, then that individual could be evaluated thoroughly. The patient could be counselled intensively and supported to take their treatment if poor adherence was found to be a problem. Management of co‐morbidities could be enhanced. Samples could be taken to evaluate for drug resistance and further investigations carried out to look for other diagnoses. Ultimately, it may be possible to use a change in biomarker status after several days to support the diagnosis of TB in those clinically diagnosed. It might be possible to conclude that those without any change in TB‐specific biomarkers might not have had TB at baseline or have drug‐resistant disease.

### 2.4 Tailoring therapy to disease severity and treatment response

In many areas of medicine, personalized precision therapy is becoming more common with treatment targeted to host genotype, disease type, disease site, disease severity, and response to treatment. Yet for programmatic reasons, almost all TB cases are given the same combination of drugs, at the same dosages and for the same duration.^18^ Most children with TB do not need 6 months of therapy and some might be successfully treated with substantially shorter durations. Early TB trials in adults demonstrated that although most patients with sputum smear‐negative TB were cured after even 2 months, an unacceptably high proportion relapsed.^29^ As it was not possible in those early studies to predict which patients might relapse, it was felt preferable to treat all patients with the minimum duration to achieve relapse free cure in >95%. This ultimately means overtreating most individuals and almost all children.

Increasingly there is recognition that different patients with different forms of TB may be appropriately treated with different drug combinations, dosages, or durations. A recently completed phase 3 clinical trial called SHINE (Shorter Treatment for Minimal Tuberculosis in Children) recruited children with minimal TB and randomized them to either the conventional 6 months of treatment or a new 4‐month treatment duration using the same drugs.^30^ In children, minimal or paucibacillary disease accounts for two thirds of all childhood TB, and so, many children would be spared the additional and unnecessary 2 months of treatment. The trial found that 4 months of treatment were not inferior to the longer treatment duration. This exciting development has led to a revision to WHO guidance,^31^ but a key challenge is to reliably define non‐severe disease. For the trial, non‐severe disease was classified as extra‐thoracic lymph node TB or pulmonary TB which was sputum smear‐negative and non‐severe on chest X‐ray. These are not easy to determine and are subject to substantial inter‐investigator variability.

A biomarker that could discriminate severe disease from non‐severe disease would pave the way for decision‐making at baseline that could be stratified, or ultimately personalized. In addition to stratifying children at baseline into different phenotypes that may benefit from different therapeutic approaches, it may also be possible to tailor treatment duration to therapeutic response. A biomarker that modelled the trajectory of response to treatment would make it possible to decide when a child has returned to a “normal” state and at that point it might be possible to stop treatment.

### 2.5 Identifying which children and adolescents will develop disease‐related morbidity

There is increasing recognition that many TB survivors suffer substantial morbidity. A fifth of children with TB meningitis die, but of survivors over half have permanent long‐term neurological impairment.^26^ Although data in children and adolescents are limited, over half of adults who have survived pulmonary TB have substantial respiratory morbidity, and those surviving TB have increased risk of death.^32^, ^33^ Although severity of disease at baseline is a strong indicator of long‐term morbidity, the reasons why some individuals develop post‐TB morbidity while others do not is poorly understood and is likely to be due to the host inflammatory response causing host tissue damage and scarring.

If it were possible to determine either at baseline or during treatment, which individuals were likely to develop morbidity, it may be possible to intervene. This might include host‐directed therapies (HDTs) at baseline to prevent future morbidity,^34^ or the early identification of those with impairment and provision of supportive therapy.

### 2.6 Using biomarkers in clinical research for the evaluation of new drugs or vaccines

Demonstrating the efficacy of new anti‐TB drugs or TB vaccines requires trial entry and exit points. Entry points are inclusion or exclusion criteria while exit points are trial outcomes. For TB disease treatment trials, the entry point is TB disease, and the exit points include cure, treatment completion, treatment failure, death, or TB relapse. For TPT trials and most vaccine trials that aim to prevent TB, the entry point is the exclusion of TB disease, with the outcome of interest being TB disease or death. If a biomarker were able to distinguish children with TB disease from those without, the ascertainment of these entry and exit points would be made much easier. In addition, if a biomarker was available that indicated children and adolescents with *Mtb* infection who were at higher risk of disease progression, then TPT trials might opt to focus only on those individuals, making the sample size required for a trial much smaller. In addition, if surrogate biomarkers were identified that served as a correlate of disease or protection, ones which indicated future disease progression or treatment failure but at a much earlier timepoint than clinical outcomes, then the duration, sample size, and cost of clinical trials of new TB drugs (for both TPT and TB disease treatment) as well as for new vaccines, could be reduced.

### 2.7 Biological insight

The development of new vaccines and new HDTs requires an insight into the biological interaction between the host and *Mtb*. The aim of most vaccines is to either prime or modulate adaptive immune responses, so that when *Mtb* is encountered, the response is more effective and can either contain or eradicate the organisms before they proliferate and causes disease. The aim of most TB HDTs is to promote helpful inflammatory processes that assist the immune response in containing or eradicating *Mtb*, while inhibiting the damaging, destructive components of the host response that either assist bacterial proliferation or cause substantial host tissue damage that leads to mortality or long‐term morbidity beyond the impact of *Mtb*.

By comparing the transcriptomic response of children who have been exposed to *Mtb* and do not progress to disease, with TB‐exposed children who do progress, it may be possible to better understand the immune response that is effective in *Mtb* containment. Vaccines that seek to prevent TB, should aim to promote those immune responses. When evaluating the impact of HDTs on TB pathogenesis, it would be informative to compare children with TB and extensive host damage with child with minimal damage. In this way, destructive inflammatory pathways might be identified that could be treated with targeted HDTs.

### 2.8 Impact of coinfections on TB susceptibility and disease progression

Children and adolescents are infected with multiple pathogens during the first two decades. Consequently, TB‐coinfections are common in high TB‐prevalence regions,^35^ and there is growing recognition that coinfections may influence TB susceptibility, natural history, and the performance of diagnostics.^36^, ^37^

Infection with a co‐pathogen may provoke an immune response which may disrupt anti‐mycobacterial immunological pathways important for controlling and containing *Mtb* infection.

Growing evidence suggests that viral coinfections such as influenza may increase susceptibility to *Mtb* infection or increase the risk of progression to disease.^37^, ^38^, ^39^, ^40^, ^41^, ^42^ Understanding the biology and immunological consequences of coinfection on *Mtb* infection and disease is important as it could impact the performance of host immune‐based TB diagnostics including transcriptomic signatures, and might also have implications for clinical decision‐making and development of vaccines and immunotherapies.^37^

TB‐HIV coinfection has been extensively studied, and it is estimated that HIV increases the risk of incident TB disease in children eight‐fold, with the risk higher in those more immunosuppressed.^43^ Understanding how HIV increases TB susceptibility in children is incompletely understood and requires pediatric‐specific studies. Comparing transcriptomic responses with TB in children living with HIV and in children without HIV may shed light on the mechanisms underlying their differing susceptibilities. In addition, the impact of HIV coinfection on the performance of transcriptomic‐based diagnostic biomarkers must be understood for them to be clinically useful in coinfected populations, and it is possible that different transcriptomic TB signatures are required for HIV‐positive and ‐negative children.

Since the COVID‐19 pandemic began in early 2020, over 540 million confirmed cases of severe acute respiratory syndrome coronavirus 2 (SARS‐CoV‐2) infection have been reported worldwide,^44^ with the true number of infections likely to be substantially higher. Countries with high TB incidence such as India and South Africa have reported tens of millions of SARS‐CoV‐2 infections to date.^44^ The pandemic has disrupted health services and TB control programs,^12^ with significant drops in case finding and treatment^6^ and predictions that this will lead to increased disease burden and mortality.^12^, ^13^, ^14^, ^15^ With their overlapping epidemiology, risk factors, and clinical presentations, studying the effects of *Mtb*‐SARS‐CoV‐2 coinfection should be a research priority.

Studies of *Mtb*‐SARS‐CoV‐2 coinfections are limited and focus on adults.^13^, ^45^, ^46^, ^47^ Their overlapping clinical presentations mean that distinguishing between SARS‐CoV‐2, *Mtb*, and *Mtb*‐SARS‐CoV‐2 coinfection can be diagnostically challenging. As with other non‐*Mtb* pathogens, when a TB diagnosis is under consideration, the detection of SARS‐CoV‐2 may exacerbate diagnostic uncertainty as current microbiological tests cannot distinguish between a colonizer, pathogen, and co‐pathogen. A biomarker that measures the host immune response could provide insight as to whether the detected pathogen is contributing to the clinical presentation or just a bystander. As with HIV coinfection, SARS‐CoV‐2 will need to be considered when developing and evaluating host‐based diagnostic biomarkers for TB.

In adults, TB disease is a risk factor for severe COVID‐19 disease and associated mortality,^48^, ^49^, ^50^, ^51^ but very few studies have considered the immunological effects of *Mtb*‐SARS‐CoV‐2 coinfection. A South African study has suggested an interaction via alterations in T‐cell function including reduced *Mtb*‐specific CD4+ cells in adults with COVID‐19 disease.^52^ Whole blood interferon‐γ responses to SARS‐CoV‐2‐peptide stimulation have been observed to be lower in adults with TB and COVID‐19 disease compared with those with COVID‐19 disease only.^53^ A recently published study of a *Mtb*‐SARS‐CoV‐2 co‐infection mouse model reported the surprising finding that chronic pulmonary *Mtb* infection protected mice from the effects of a SARS‐CoV‐2 challenge, with an associated expansion of pulmonary T and B cell subsets observed. SARS‐CoV‐2 infection did not, however, affect *Mtb*.^54^ Further immunological studies of *Mtb*‐SARS‐CoV‐2 interactions are needed to understand the potential impact of the COVID‐19 pandemic on TB susceptibility and should include pediatric and adolescent populations. Comparisons of transcriptomic responses to *Mtb* in SARS‐CoV‐2 infected and uninfected patients will highlight immunological pathways for further functional studies.

## 3 THE ROLE OF TRANSCRIPTOMICS

Induction of tissue and immune responses against *Mtb* and the intracellular signaling between immune cells trigger a biochemical chain of events, which leads to the production of molecules needed for defence against *Mtb*. Untargeted host molecular profiling (transcriptomic, proteomic, and metabolomic) in tissues from the site of disease using high‐throughput methodologies can be employed to shed light on biological processes and identify key biological molecules. These methods can help identify biomarkers and provide an understanding of the dynamics of infection and inflammation, for example, through highlighting the key pathways involved in the biological process. Studying the host transcriptome in the lungs^55^ or other disease sites^56^ can elucidate local pathogenic characteristics of the disease and the host immune response at the tissue or cellular level. Host transcriptomic analysis of bronchoalveolar lavage (BAL) or sputum samples has revealed strong type I/II interferon‐mediated cytokine responses and T‐cell activation in adults with TB.^57^

Due to accessibility, minimal invasiveness in collection, and the key role peripheral blood and its compartments play in host defense and immunity, peripheral blood has become the focus tissue for host transcriptomic studies in child and adolescent TB. This follows work done in adult studies which first established that immune changes associated with pulmonary disease can be identified and quantified in RNA derived from peripheral blood.^58^ In this review, we focus on transcriptomic profiling of whole blood cells and peripheral blood mononuclear cells (PBMC) in the context of *Mtb* infection and disease.

As RNA is sensitive to degradation, which can hamper the quantification of results, specific RNA stabilizing systems are used for sample collection, shipping and storage that allow preservation of RNA. Different RNA stabilizing blood‐sampling systems may introduce differences in the downstream quantification results, with thousands of genes being reported as significantly differentially expressed (SDE) between RNA stabilizing reagents. This needs to be taken into consideration in study design and meta‐analyses.^59^ Subsequently, fine‐tuned protocols for RNA purification can ensure the quality, integrity and yield of isolated RNA, along with minimization of potential DNA contamination.^60^ Recent studies have shown in vitro whole blood stimulation with *Mtb* antigen peptides, which has been used in proteomic biomarker discovery studies, can unmask transcriptomic signals that are not detectable in unstimulated samples^61^ or enhance the diagnostic potential of single gene markers in high burden settings.^62^

In terms of sample volume, although blood–volume dependent reduction in gene levels has been reported, transcriptomic profiling can be achieved with small volumes of blood, which is particularly important in young children. High quality RNA‐Sequencing results have been reported in neonates using volumes as low as 0.5 mL of peripheral venous or arterial blood.^63^

## 4 TRANSCRIPTOMICS METHODOLOGY

### 4.1 Methods for RNA quantification

The “candidate gene” approach focuses on measuring expression for small numbers of genes and can be used when the genes of interest are already known. Reverse transcription quantitative polymerase chain reaction (RT‐qPCR) is a sensitive, accurate, highly reproducible method able to detect very small amounts of RNA. It is considered a benchmark technology and forms the basis of various nucleic acid identification platforms with bedside use. Reverse transcription as a methodological step is shared between most protocols for RNA quantification. The fluorescence emitted at the end of each cycle of PCR is used for the estimation of the quantity of the starting material.^64^ RT‐qPCR has enabled multiple scientific breakthroughs and can now be used to simultaneously detect and quantify multiple nucleic acids through multiplexing.^65^, ^66^ The relatively new NanoString nCounter gene expression system first reported in 2008 (Nanostring Technologies, WA, USA) has introduced a new method for targeted RNA quantification based on color‐coded probe pairs' ability to hybridize with complementary mRNA and fluorescence.^67^ It can be used for quantification of custom genes or inventoried gene panels according to application area or biological process of interest.^68^

Transcriptome‐wide profiling methods allow for systematic analysis of thousands of RNA molecules simultaneously in a high‐throughput manner and provide a global quantitative profile of the cell or tissue of interest, without the need of a pre‐existing research hypothesis. Microarray technology exploits the principles of specific hybridization between two DNA strands and the emission and detection of fluorescence proportional to the amount of nucleic acid that is bound. For gene expression quantification, it involves reverse transcription of the isolated RNA to cDNA, followed by amplification and labelling with a fluorescent dye. Subsequently, the dyed cDNA is hybridized to the array under specific conditions, which permit its binding to complementary sequences already printed on the array, followed by washing to eliminate non‐specific binding events, and insertion into a scanner that both excites the fluorescent dyes and records the emitted intensity. The fluorescence signal emitted by the probes reflects the abundance of the corresponding RNA transcript in the sample. Prior knowledge of the position of reference transcripts allows for relative quantification of the transcripts bound to specific probes.^69^, ^70^ Gene expression microarrays have been used as the discovery tool in many TB transcriptomic studies,^71^, ^72^ and although superseded by RNA sequencing (RNA‐Seq), they are still being extensively used due to lower cost, years of standardization, and less intensive bioinformatic analysis needed.

Over the past decade, RNA‐Seq has become an indispensable and popular tool for transcriptome‐wide analysis with many applications in studying TB host response as it is independent from pre‐existing sequence information. However, of the total RNA in a cell, 80% is ribosomal RNA (rRNA), 15% is transfer RNA (tRNA), leaving only 5% as mRNA and all the other RNA forms.^73^ To focus on the RNA molecules of interest, RNA‐Seq libraries are prepared using either polyA+ selection (mRNA enrichment) or rRNA depletion. rRNA depletion allows the detection of more transcripts including non‐coding RNA (ncRNA), small nucleolar RNA (snoRNA), and small nuclear RNA (snRNA). A comparison between the two methods has shown that although rRNA depletion captured more unique transcriptome features, for blood‐derived RNAs, 220% more reads would have to be sequenced to achieve the same level of exonic coverage in the rRNA depletion method compared with the polyA+ selection approach.^74^ Globin transcript depletion is another critical step to obtaining data suited for blood transcriptome analysis. Total RNA from whole blood contains a large portion of globin transcripts, which originate from red blood cells and account for 80–90% of total transcripts.^75^ These affect the quality and accuracy of gene expression profiling and mask the quantification of genes with low expression levels. Next Generation Sequencing (NGS) technology is based on detecting and recording light that is emitted when a complimentary nucleotide is added to a particular fragment of cDNA. The light detected will determine the identity of the nucleotide (“base calling”) and subsequently the sequence of the whole “read” in single base‐resolution. The reads are either mapped bioinformatically to a reference genome or assembled de novo to produce the transcriptome, a base‐resolution expression profile. Read mapping allows for the quantification of RNA and providing abundance estimates.^76^ RNA‐Seq has revolutionized the field of transcriptomics, also allowing discovery of novel transcripts, alternative splicing, the detection of gene fusion events and allele‐specific expression. RNA‐Seq also permits simultaneous sequencing of pools of transcripts that may come from different organisms that coexist in the same environment, termed metatranscriptomics.^77^, ^78^

### 4.2 Analytical approaches for transcriptome‐wide profiling

The quality control, pre‐processing and analysis steps for microarrays are mostly standardized, while RNA‐Seq data analysis pipelines, which are more complex and computationally demanding, can consist of a greater variety of steps and tools. The abundance per feature per sample is the input for differential expression analysis for both quantification methods. After quality assessment and exclusion of poor‐quality samples, normalized microarray expression values are used for downstream analysis. For RNA‐Seq, sequence reads need to be adapter‐ and quality‐trimmed, and then aligned either to the human genome or transcriptome.^79^ Features then are quantified, with low abundance features filtered and followed by normalization processes to account for biases, noise, and sequencing depth variation. Subsequently analytical approaches follow according to the biological and clinical questions that are being addressed (i.e., differential gene expression analysis and alternative splicing analysis). Both microarray and RNA‐Seq differential expression analysis workflows are followed by multiple testing corrections to control for false positive errors.

The data analysis workflow is quite different for studies intending to discover diagnostic vs. mechanistic transcriptional signatures of disease. The one shared component is a set of initial algorithms to identify gene sets associated with different disease states, termed differentially expressed genes. For biomarker discovery, feature selection methods are employed to identify the marker or the combination of markers that minimize the classification error or maximize the accuracy of classification for patient subgroups, while eliminating noise and redundant genes. Feature selection methods are divided into filter, wrapper, and embedded methods.^80^ Filter methods select a feature subset from the original dataset by evaluating the relation between each input variable and the target variable (e.g., statistical methods or feature importance methods). They are usually used as a pre‐processing step, followed by a machine learning algorithm. The wrapper methods search for a well‐performing subset of features by training a model on it. These iterative methods add (e.g., forward selection) or remove (e.g., backward elimination) features based on the performance of the trained model. The embedded methods combine the advantageous aspects of both filter and wrapper methods since they perform feature selection as part of the classifier construction. Many of these methods have an inbuilt penalization function to shrink the coefficients of the least important features toward zero (e.g., Least Absolute Shrinkage and Selection Operation regression) and keep in the model the most relevant features. Appropriate partitioning of datasets, representing samples, into training and test sets, and the use of iterative resampling methods (e.g., cross‐validation) can minimize overfitting, while independent validation datasets can ensure the robustness and generalizability of the findings. To evaluate the performance of biomarker models, different point estimates and interval metrics are typically used for diagnostic tests.^81^, ^82^, ^83^ Results are benchmarked against a “gold standard” diagnosis, which poses a particular challenge for pediatric TB. However, for binary output case–control studies the minimal set of point estimates reported are as follows: (a) the area under the operating receiver characteristic curve (AUC), (b) sensitivity, which reflects the probability of test being positive with disease present and (c) specificity, which reflects the probability of being test negative with disease absent. In prospective recruitment studies, the positive predictive value (PPV), which reflects the probability of a patient having the disease when the test is positive, the negative predictive value (NPV), which reflects the probability of a patient not having the disease when the test is negative and likelihood ratios are reported in addition.^84^ Confidence intervals are calculated to measure the reliability of the estimates. For case–control studies, the ratio of gold standard positive and negative individuals does not reflect the real prevalence of the disease in a community or hospital setting, as in observational studies. Given the dependency of NPV/PPV on the prevalence of the disease in the population, it is important to provide estimates of these values specific to scenarios in which such a diagnostic test would be applied. In this case, prevalence can be interpreted as “the probability before the test is carried out that the subject has the disease.”^82^

### 4.3 Understanding TB biology

Apart from biomarker discovery, interpreting differential expression results in terms of higher order biological processes or molecular pathways is a key outcome in transcriptomic analysis. One of the most commonly used resources is gene ontology (GO) databases, which annotate genes according to a dictionary of annotation terms, to identify the terms that are over‐represented or enriched.^85^ Another commonly used annotation database is the Kyoto Encyclopedia of Genes and Genomes (KEGG), a curated database of molecular pathways and disease signatures.^86^ Ingenuity Pathway Analysis (IPA‐ QIAGEN) provides a series of different functionalities, allowing for the identification of significantly enriched canonical pathways, network analysis, and upstream regulating molecules.^87^ In comparison to methods using overlap statistics such as the cumulative hypergeometric distribution to identify whether a group of differentially expressed genes is enriched for a pathway or ontology term, a different method can be used termed Gene Set Enrichment Analysis (GSEA) which considers all of the genes in an experiment, rather than only those above specific cut‐offs.^88^ There are different tools for performing GSEA analysis including MSigDB,^89^ g:Profiler,^90^ and DAVID.^91^

Understanding the cellular composition of bulk tissues is critical to investigate the underlying mechanisms of many biological processes. Molecular profiling using bulk RNA‐Seq in heterogeneous tissues, such as blood, is confounded by the relative proportions of different cell types in the tissue. Single cell RNA‐Seq data is quickly becoming the “gold standard” technique for cell specific expression profiles but is an expensive and data‐analysis intensive technique. Flow cytometry is also widely used to estimate cell‐type composition in bulk tissue experimentally and data on cell fractions obtained can inform differential gene expression analysis and allow model adjustment for cell fractions. However, flow cytometry requires isolation of cells, laboratory equipment, and additional sample sacrifice.

When flow cytometry or single cell RNA‐Seq data are not available, cell‐type specific gene expression profiles and sample‐specific cell type proportions can be estimated from bulk gene expression data using computational deconvolution methods also termed *in silico* deconvolution methods. These methods are usually based on matrix factorization, which is employed to deconvolve a matrix of gene expression profiles from bulk gene expression data into two matrices, one for the cell‐type proportions for each sample and another containing the gene expression profiles for each cell type. Some of the developed algorithms performing partial deconvolution need either cell‐specific gene expression profiles or cell‐type proportions as input, and use marker genes or regression techniques to estimate the matrix of interest.^92^, ^94^ Other methods are able to estimate both matrices using only bulk gene expression data.^95^

## 5 BIOMARKERS IN CHILD AND ADOLESCENT TB

Parsimonious gene expression signatures that have the potential to be measured at a clinical setting, and particularly in a low resource clinical setting in TB endemic countries, can assist in successfully addressing the multiple challenges faced in the field of child and adolescent TB, and in particular disease diagnosis, prognosis, and treatment decision‐making. A number of research studies have been published presenting results from gene expression analysis for the identification of diagnostic, prognostic, and treatment response biomarkers (Table 1).

**TABLE 1:** Studies that have used transcriptomic approaches in child and adolescent TB, presenting original patient recruitment data and analysis

First author	Year published	Country ‐Population	Description of study	Number of children/adolescents analyzed (original data)	Main findings of study
Verhagen^97^	2013	Venezuela	Discovery (microarray) and validation (RT‐qPCR) of gene expression signature to distinguish TB from children with *Mtb* infection, healthy controls and non‐TB pneumonia (validation only)	9 TB patients, 29 with *Mtb* infection, 25 healthy controls and 18 non‐TB pneumonia	A 116‐gene signature for TB vs. *Mtb* infection and healthy controls with an average prediction error of 11%, and a 9‐gene signature for TB vs *Mtb* infection. An optimized set of 5 genes had 4% false positive rates for healthy controls and 11% for non‐TB pneumonia cases.
Dhanasekaran^101^	2013	India	Whole‐blood mRNA from 210 children was examined by dcRT‐MLPA for the expression of 45 genes	13 children with TB disease, 90 with *Mtb* infection, 107 uninfected controls	A single gene discriminated between TB and *Mtb* infection (AUC 0.78). A 5‐gene signature discriminated TB disease from controls (AUC 0.92). An 11‐gene signature distinguished *Mtb* infection from controls (AUC 0.72).
Anderson^99^	2014	South Africa, Malawi, Kenya	Discovery and validation of gene expression biomarkers from microarray data to distinguish TB from *Mtb* infection and TB from Other Diseases	149 children with culture‐confirmed TB, 44 with unconfirmed TB, 71 with *Mtb* infection and 139 with Other Diseases	A 51‐trasncript signature for TB vs Other Diseases and a 42‐transcript signature for TB vs *Mtb* infection with sensitivity of 82.9% and specificity of 83.6% in the independent validation cohort.
Li^62^	2015	China	Quantification by RT‐qPCR of 7 genes in PBMC after ESAT‐6 stimulation in children with PTB, EPTB and healthy controls	39 children with TB (3 smear culture positive and 26 culture negative) and 25 healthy controls	The expression of *IL‐9* separated children with TB vs healthy controls with an AUC of 0.92 after ESAT‐6 stimulation.
Wang^102^	2015	China	RT‐qPCR was used to quantify miR‐31 expression in PBMCs from children with TB and healthy controls	65 children with TB and 60 healthy controls	The expression of miRNA‐31 distinguished children with TB from healthy controls with sensitivity of 98.5% and a specificity of 86.7%.
Zak^110^	2016	South Africa	Discovery using blood RNA‐Sequencing, and validation (RNA‐Sequencing and RT‐qPCR) of a signature to predict progression of *Mtb* infection to disease in adolescents (12–18 years)	46 progressors and 107 matched controls	A 16‐gene signature for TB progression which had sensitivity of 53.7% and specificity of 82.8% in the 12 months preceding TB in independent South African and Gambian cohorts.
Fletcher^137^	2016	South Africa	Infants were vaccinated with BCG at birth and followed for 2 years. Blood was collected at 10 weeks. Host responses from the 10‐week samples were compared between those who developed TB disease within 2 years and controls who remained healthy.	5726 infants were recruited. 29 cases of confirmed TB were compared to 110 controls (55 household controls and 55 community controls).	Gene expression analysis did not show a difference between cases and controls.
Jenum^104^	2016	India	Targeted analysis of transcriptional immune biomarkers in *Mtb*‐antigen stimulated whole blood using dcRT‐MLPA	88 children with intra‐thoracic TB (6 months ‐ 15 years); 40 culture‐confirmed, 48 unconfirmed and 39 asymptomatic	An 8‐gene biomarker signature separated children with TB from asymptomatic siblings (AUC 0.88) in stimulated blood. 12 genes were found associated with clinical groups toward culture‐positive TB or toward a decreased likelihood of TB disease on the TB disease spectrum.
Zhou^103^	2016	China	Identification of circulating miRNAs that can differentiate between TB and healthy controls	14 culture‐positive TB cases, 14 culture‐negative TB cases and 25 children with TB and 21 healthy controls for validation	An 8‐miRNA signature provided 95.8% sensitivity and 100% specificity for the discrimination of children with TB vs uninfected healthy controls
Gjoen^100^	2017	India	Selection and optimization of 2 signatures for TB vs asymptomatic household controls, and other symptomatic non‐TB cases, from a set of 198 genes using dcRT‐MLPA.	71 TB cases (36 definite/35 probable) and 36 asymptomatic household controls, and 26 symptomatic non‐TB cases.	A 7‐and a 10‐transcript signature with AUC of 0.94 in separating TB‐cases from symptomatic non‐TB cases regardless of culture status, and 100% sensitivity for definite TB.
Hemingway^121^	2017	South Africa	Longitudinal microarray blood gene expression analysis in children with TBM and comparison with children with PTB	9 children with TBM, (4 timepoints) and 9 healthy controls; 13 children with TBM and 28 with PTB.	Reduced abundance of 68% of SDE genes in TBM vs healthy controls. The difference in abundance was less in PTB than in TBM.
Rohlwink^122^	2019	South Africa	RNA‐Sequencing on whole blood as well as on ventricular and lumbar cerebrospinal fluid of pediatric patients treated for TBM	20 TBM cases 20, 7 Non *Mtb* infection controls, and 24 healthy controls	2230 genes were SDE in TBM cases vs healthy controls in blood, and 312 genes were SDE in ventricular CSF in TBM vs infection controls. TB disease processes differ between the periphery and the central nervous system, and within brain compartments.
Penn‐Nicholson^113^	2020	South Africa, The Gambia, Ethiopia, Peru, Brazil	Identification of a parsimonious signature from the RNA‐Sequencing Zak et al. data using RT‐qPCR data, and subsequent validation as a signature for diagnosis, progression and treatment response.	46 progressors and 107 matched controls (Adolescent cohort study)	A 6‐gene transcriptomic signature of TB disease risk, diagnosis and treatment response
Tornheim^116^	2020	India	Longitudinal RNA‐Sequencing from whole blood in cases during treatment and controls for the identification of differentially expressed genes.	16 TB cases and 32 TB‐exposed controls	A 71 gene signature for TB diagnosis and a 25 gene signature for treatment response
Johnson^138^	2021	India	Performance of TB gene signatures in malnourished individuals (including children) with TB and *Mtb* infection	23 severely malnourished individuals with TB and 15 severely malnourished TST positive household contacts	4913 significant differentially expressed protein coding genes in TB vs *Mtb* infection in malnourished individuals; 56.9% of the genes overlap with the 45 TB gene signatures included on the paper.

Abbreviations: AUC, area under the curve; CSF, cerebrospinal fluid; dcRT‐MLPA, dual colour reverse transcription multiplex ligation dependent probe amplification assay; EPTB, extrapulmonary TB; PBMC, peripheral blood mononuclear cell; PTB, pulmonary TB; RT‐qPCR, reverse transcription quantitative polymerase chain reaction; SDE, significantly differentially expressed; TB, tuberculosis; TBM, TB meningitis; TST, tuberculin skin test.

### 5.1 Transcriptomics as a diagnostic tool

The field of infectious disease diagnostics has embraced molecular tools that profile the host response and can enhance disease diagnostic pipelines, particularly when the detection of the pathogen of interest is challenging, as in pediatric TB.^96^ In clinical practice, a gene signature measured in blood that can distinguish pediatric TB from other diseases with similar presentation to TB would be of great value in evaluating symptomatic patients presenting to medical services with symptoms of TB.

Two studies to date have discovered diagnostic gene signatures specific for pediatric TB in a hypothesis‐free transcriptome‐wide manner. Verhagen and colleagues in 2013 published the first microarray profiling study for pediatric TB biomarker identification in Warao Amerindian children.^97^ A signature of 116 genes identified by the random forest algorithm separated 9 TB cases from 9 with *Mtb* infection and 9 healthy controls in the training set, which was then subsequently validated in publicly available adult datasets.^72^, ^98^ Following random forest bootstrapping, the list was reduced to 10 genes that was validated using RT‐qPCR in the discovery cohort, and in 20 children with *Mtb* infection, 16 healthy children, and 18 children with non‐TB pneumonia. Further decision tree analysis indicated that five genes classified 78% of the TB cases correctly with a 0% false positives for the *Mtb* infection group, 4% for the healthy controls, and 11% for the non‐TB pneumonia cases.

**FIGURE 2:**
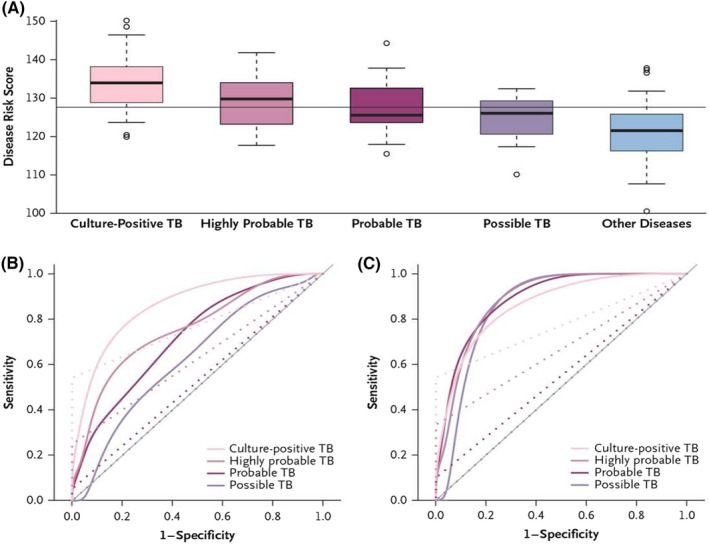
Risk Scores and Sensitivity and Specificity in the Kenyan Validation Cohort, According to Diagnostic Group. Panel A shows the risk scores for tuberculosis according to study group, calculated with the use of a 51‐transcript signature applied to the independent Kenyan validation cohort, in which culture‐positive tuberculosis was reported in 35 patients, diseases other than tuberculosis were reported in 55 patients, and culture‐negative tuberculosis was reported as highly probable in 5 patients, probable in 19 patients, and possible in 17 patients. The bar within each box indicates the median score, the bottom and top of the box indicate the interquartile range, the bars below and above the box are at a distance of 0.8 times the interquartile range from the upper and lower edges of the box, and the circles indicate outliers; the horizontal line across the graph indicates the mean score. Panel B shows smoothed receiver‐operating‐characteristic (ROC) curves for the sensitivity and specificity of the risk score (solid lines) and the Xpert MTB/RIF assay (dotted lines). Panel C shows ROC curves based on an adjusted analysis in which the actual prevalence of disease was assumed to be 80% among patients in whom the disease was highly probable, 50% among those in whom it was probable, and 40% among those in whom it was possible. From Anderson and colleagues. New Eng J Med 2014; 370: 1712–23

In 2014, Anderson and colleagues described the discovery of transcriptional signatures for distinguishing culture‐confirmed TB from diseases other than TB in a multicohort pediatric population (<15 years of age), comprising of HIV‐positive and HIV‐negative children with symptoms and clinical findings that were suggestive of TB, using microarray analysis of host blood.^99^ Patient data from South Africa and Malawi were combined into a discovery set (80% training and 20% test). SDE transcripts were subjected to feature selection using elastic net that resulted in a 51‐transcript signature with abundance combined into a Disease Risk Score. The 51‐transcript signature had a sensitivity of 82.9% (CI_95%_ 68.6–94.3), and a specificity of 83.6% (CI_95%_ 74.6–92.7) in an independent validation cohort from Kenya (Figure 2). The sensitivity exceeded that of the Xpert MTB/RIF assay which was 54.3% (CI_95%_ 37.1–68.6). Additional analysis provided estimates for sensitivity in the culture‐negative groups of highly probable, probable, and possible TB. Using a similar discovery pipeline, a 42‐transcript signature discriminated confirmed TB from *Mtb* infection with sensitivity of 94.3% and specificity of 100.0% in the independent validation cohort from Kenya.

In 2017, Gjoen and colleagues employed a dual color reverse transcription multiplex ligation dependent probe amplification assay (dcRT‐MLPA) to assess the performance of 198 genes in a training set, comprising 47 pediatric TB cases (19 definite and 28 probable) and 36 asymptomatic household controls, and identified a 7‐ and a 10‐transcript signature by using a combination of logistic regression and LASSO for feature selection.^100^ The 10‐transcript signatures had an AUC of 0.94 (CI_95%_ 0.88–1.00), correctly classifying 22 of 24 TB cases and 23 of 26 symptomatic non‐TB cases, corresponding to a sensitivity of 91.7% (CI_95%_ 71.5–98.5) and a specificity of 88.5% (CI_95%_ 68.7–96.9). The 7‐transcript signature also provided an AUC of 0.94 (CI_95%_ 0.88–1.00) but classified correctly only 21 of 26 symptomatic non‐TB cases.

A multicohort meta‐analysis by Sweeney and colleagues in 2016 included 14 datasets containing 2572 samples from 10 countries from both adult and pediatric patients and identified a 3‐gene signature reporting an AUC for discriminating TB from other diseases of 0.84 (CI_95%_ 0.80–0.95) and from *Mtb* infection 0.88 (CI_95%_ 0.84–0.92). For the comparison of TB vs. other diseases in the previously reported pediatric datasets from South Africa/Malawi the Sweeney 3‐gene signature had sensitivity of 68.5% and specificity of 74.0% and a sensitivity of 77.1% and specificity of 87.5% in the Kenyan dataset.^99^ The metrics for the pediatric dataset from South Africa/Malawi are lower in comparison to the global metrics reported for TB vs. other diseases including the adult datasets (sensitivity 81%, specificity 74%), highlighting the need for pediatric‐specific signatures or the inclusion of genes that boost the performance in the pediatric datasets to account for the different nature of the disease, the different host response and the fact that pediatric TB needs to be discriminated from a different set of other diseases than adult TB in clinical practice.

A larger number of studies have been published that focus on discriminating TB from healthy uninfected controls and children with *Mtb* infection (Table 1). Although important as a proof‐of‐concept, this discrimination has less clinical utility. As this is a disease vs. healthy comparison, the host response is more perturbed, and the groups can be discriminated more easily and with fewer genes. In 2013, Dhanasekaran and colleagues identified a 5‐gene signature that can discriminate children with TB from healthy controls and an 11‐transcript signature that can discriminate children with TB from children with *Mtb* infection, using dcRT‐MLPA in a pediatric population in India.^101^ Wang and colleagues in 2015 reported that a single micro RNA (miRNA) can distinguish children with TB from healthy controls with sensitivity of 98.5% and a specificity of 86.7%,^102^ while in 2016 Zhou and colleagues identified a 8‐miRNA signature that had 95.8% sensitivity and 100% specificity for the discrimination of children with TB vs uninfected healthy controls.^103^ Antigen specific stimulation has also been employed prior to gene expression quantification. Li and colleagues reported that a single gene (IL‐9 mRNA) can separate children with TB vs. healthy controls with an AUC of 0.92 after ESAT‐6 stimulation,^62^ while Jenum and colleagues reported that an 8‐gene biomarker signature separated children with TB from asymptomatic siblings in stimulated blood with an AUC of 0.88.^104^

It is important to put these novel transcriptomic signatures in the context of the tools that are currently used to diagnose TB in children and compare performance. In a study of children with presumptive TB who were living with HIV, chest X‐ray features that were consistent with TB provided a sensitivity of 71.4% and specificity of 50% when comparing confirmed TB against unlikely TB.^105^ A systematic review and meta‐analysis of the accuracy of IGRAs to discriminate children with confirmed TB disease from children with other diseases demonstrated a sensitivity of 75% and a specificity of 66%.^106^ Xpert MTB/RIF in sputum demonstrated a sensitivity of 64.6% and specificity of 99% in a systematic review and meta‐analysis of performance against a microbiological (culture) reference standard. However, against a composite reference standard (including children diagnosed both clinically and microbiologically), the sensitivity was 19.7% with a specificity of 100%.^107^ Finally, in 2022, the WHO revised their child and adolescent TB guideline and, in the operational handbook accompanying the guideline, suggested a clinical decision‐making algorithm. The sensitivity of this algorithm (against a composite reference standard) was 85% with a specificity of 37%.^108^

### 5.2 Transcriptomics as a prognostic tool

**FIGURE 3:**
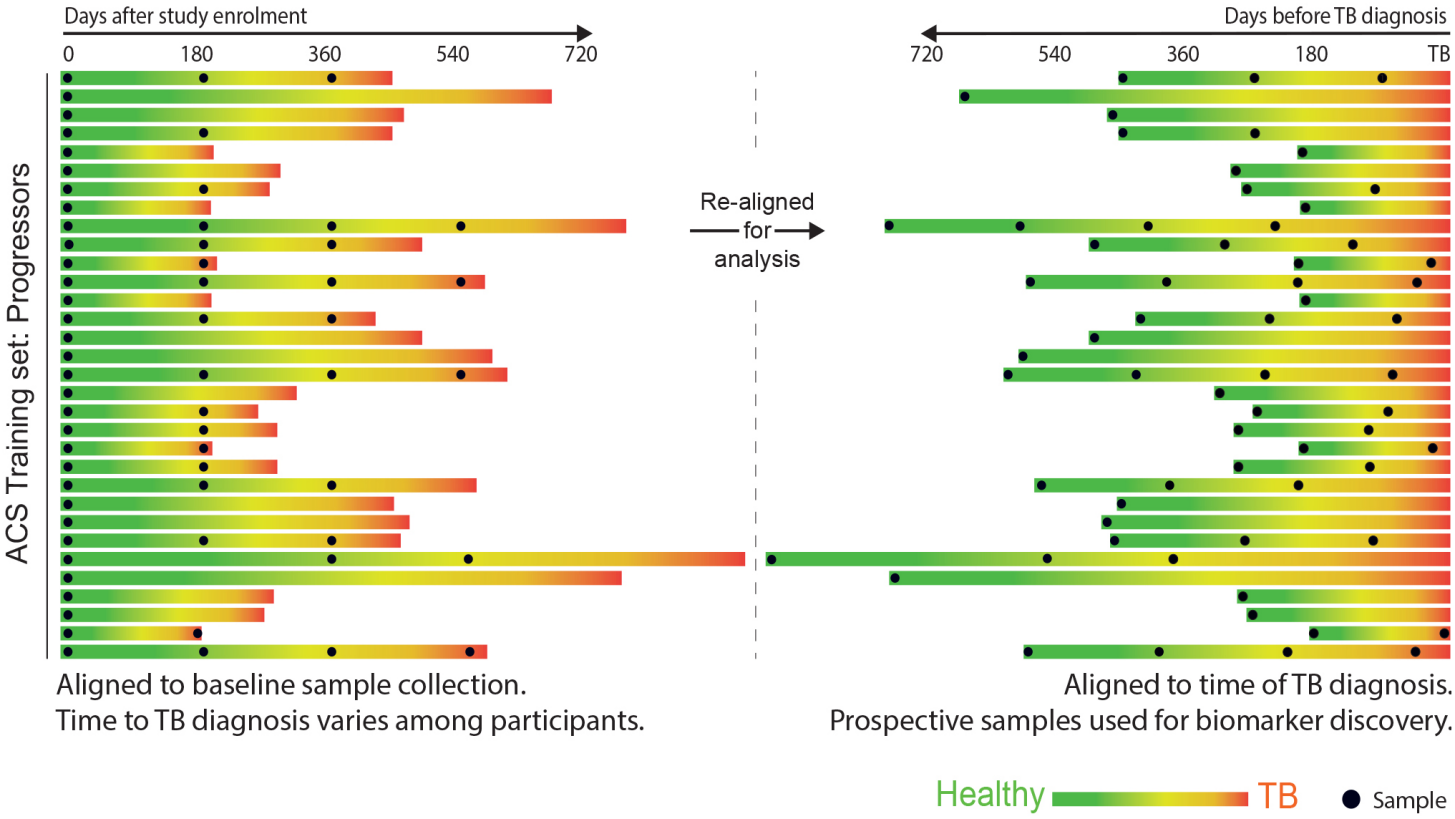
Strategy for discovery and validation of the tuberculosis risk signature. Synchronization of the adolescent cohort study training set in terms of the clinical outcome. To ensure optimal extraction of a tuberculosis risk signature from the adolescent cohort study training set, the timescale of the RNA‐Sequencing dataset was realigned according to tuberculosis diagnosis instead of study enrolment, allowing gene expression differences to be measured before disease diagnosis. Each progressor within the adolescent cohort study training set is represented by a horizontal bar. The length of the bar represents the number of days between study enrolment and diagnosis with active tuberculosis. During follow‐up, each progressor transitioned from an asymptomatic healthy state (green) to pulmonary disease (red). The left graph shows alignment of PAXgene sample collection (black points) with respect to study enrolment. The right graph shows alignment of PAXgene sample collection with respect to diagnosis with active tuberculosis, for use in analysis. From Zak and colleagues. A blood RNA signature for tuberculosis disease risk: a prospective cohort study. Lancet 2016; 387: 2312–22

A test that identifies children and adolescents who are at greater risk of disease progression would transform TB control by enabling targeted preventive therapy for the population at risk in high‐burden settings. While several studies have identified signatures in adults,^109^ few have included children or adolescents. In 2016, Zak and colleagues reported discovery of a 16‐gene signature by blood RNA‐Seq profiling in a cohort of 153 South African adolescents with *Mtb* infection, using samples collected at different timepoints prior to TB diagnosis, which was able to discriminate between TB progressors from non‐progressors (Figure 3).^110^ The prognostic performance of the signature was dependent on the time interval between sampling and disease diagnosis. Signature performance was better when measured in samples collected more proximal to disease diagnosis than in samples collected at distal time points. At 6 months prior to diagnosis, the AUC was 0.79 (CI_95%_ 0.76–0.82). Subsequent validation using RT‐qPCR, in the adolescent cohort, and in independent Gambian, Ethiopian, and South African adult cohorts replicated the findings. As the genes in the signature overlap with signatures of TB disease, it is evident that progressors have a host response similar to that of TB disease, albeit at lower magnitude, long before diagnosis is established. It is therefore most likely that such signatures identify incipient or sub‐clinical disease, as recently confirmed in the CORTIS study.^111^ A subsequent study developed a more parsimonious signature in the adolescent cohort based on 6 genes (RISK6),^112^, ^113^ which was further evaluated in an additional multi‐country adult study for TB diagnosis.^114^ As for diagnostic tools, it is important to compare these transcriptomic prognostic signatures with other tools used for clinical decision‐making. Although children <5 years with positive IGRA or TST tests are at ~8 times greater risk of TB disease progression than those with negative IGRA tests, only about 20% of children of this age with a positive IGRA/TST will progress to disease.^21^ A positive test therefore may have relatively high sensitivity, but low specificity.

### 5.3 Treatment response

**FIGURE 4:**
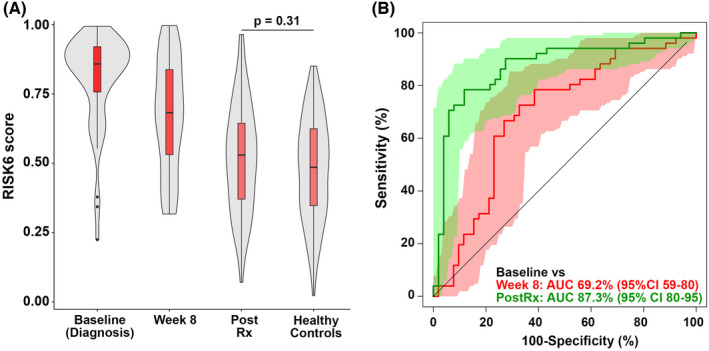
Diagnostic performance and treatment monitoring in South American cohorts. (A) Comparison of RISK6 signature scores in TB cases at baseline, Week 8 after treatment initiation and after treatment completion (Post Rx). Also shown are the RISK6 signature scores in healthy controls from Brazil. Horizontal lines depict medians, the boxes the IQR, and the whiskers the range. Violin plots depict the density of data points. The *P*‐value, computed by Mann–Whitney U test, compares RISK6 signature scores after treatment completion with those in controls. (B) ROC curves depicting performance of RISK6 for discriminating between baseline samples from TB cases and samples collected 8 weeks after treatment initiation, or upon completion of TB treatment (Post Rx). From Penn‐Nicholson and colleagues. RISK6, a 6‐gene transcriptomic signature of TB disease risk, diagnosis, and treatment response. Sci Rep 2020; 10: 8629

Currently available tests have very low accuracy for monitoring TB treatment response and predicting failure or relapse in pulmonary TB even in adults.^115^ To improve disease outcomes, we need better biomarkers to identify appropriate responses to treatment, that will allow us to identify treatment failure early and enable shortening treatment. It has been shown that the RISK6 signature tracks treatment response in adults,^113^ and has been evaluated in monitoring treatment response in a multi‐country cohort (Figure 4),^114^ but studies in children are limited.

In 2020, Tornheim and colleagues identified a 25‐gene list of differentially i have spotted expressed genes for treatment response in children, in 16 pediatric Indian TB cases and 32 age‐ and sex‐matched TB‐exposed controls. The 25‐gene treatment response list was evaluated against adult datasets with AUCs of 0.90 (CI_95%_ 0.74–1.00), 0.72 (CI_95%_ 0.85–0.94), and 0.50 (CI_95%_ 0.36‐0.63).^116^

### 5.4 Mtb‐SARS‐CoV‐2 coinfections and how they impact transcriptomic biomarkers

COVID‐19 and TB disease are both primarily respiratory pathogens and cause overlapping clinical syndromes in children and adolescents. A clinically useful transcriptomic diagnostic biomarker will need to discriminate between these two infections as well as other common respiratory pathogens. A recently published large scale systematic review and meta‐analysis of COVID‐19 transcriptomic signatures investigated their commonality with the transcriptome in TB disease. The authors identified 35 eligible COVID‐19 signatures associated with disease severity, derived from whole blood, PBMCs, and BAL fluid. Expression of these signatures was profiled on whole blood RNA‐Seq samples from 3 independent adult TB cohorts, comprising of patients with TB disease, *Mtb* infection, including those progressing (“progressors”) and not progressing (“non‐progressors”) to TB disease over the next 2 years, and uninfected controls. Twenty COVID‐19 gene signatures were significantly associated with higher “COVID‐19 risk scores” in TB disease patients and progressors, compared with non‐progressors (*P* < 0.005). By comparison, an influenza signature score was zero across the cohort, regardless of TB status. From profiling 20 single‐cell (sc)RNA‐Seq immune cell population signatures, the authors found that neutrophil and monocyte COVID‐19 signatures generated the highest COVID‐19 risk scores in TB disease and progressors, in contrast to adaptive immune cell signatures which were higher in non‐progressors. The authors also performed a meta‐pathway enrichment analysis using whole blood transcriptomic data from one of the adult TB cohorts, a COVID‐19 whole blood scRNA‐Seq dataset plus an influenza viral control cohort. In contrast to the influenza group, the COVID‐19, TB disease and TB progressor groups were all strongly enriched for interferon‐γ and tumor necrosis factor response pathways. Of the top 100 enriched pathways, just one (Hallmark of mTORC signaling) was unique to COVID‐19 and absent in TB and influenza.^117^ Not only does this study highlight the great potential for transcriptomic studies to elucidate underlying biological mechanisms of infectious diseases, but this work also suggests that pre‐existing TB diagnostic signatures may perform less well in the context of COVID‐19 due to shared immune responses. Thus, when discovering and developing future diagnostic transcriptome‐based biomarkers, it will be important to include COVID‐19 disease as a comparator group. In addition, the impact of SARS‐CoV‐2 coinfection on the performance of pre‐existing and future pediatric biomarkers should be determined, to enable them to be interpreted and deployed in real‐world settings where *Mtb*‐SARS‐CoV‐2 coinfection is likely to be common. Pediatric immune responses to SARS‐CoV‐2 and *Mtb* infection differ from adults, and therefore, it is imperative that pediatric‐specific studies are undertaken.

## 6 USING TRANSCRIPTOMICS TO UNDERSTAND CHILD AND ADOLESCENT TB BIOLOGY

Recent blood gene expression profiling studies have highlighted differences between adult and childhood TB in terms of pathways involved in host response during infection and disease and progression of infection to disease.

### 6.1 Children with TB vs children with other diseases vs well children

**FIGURE 5:**
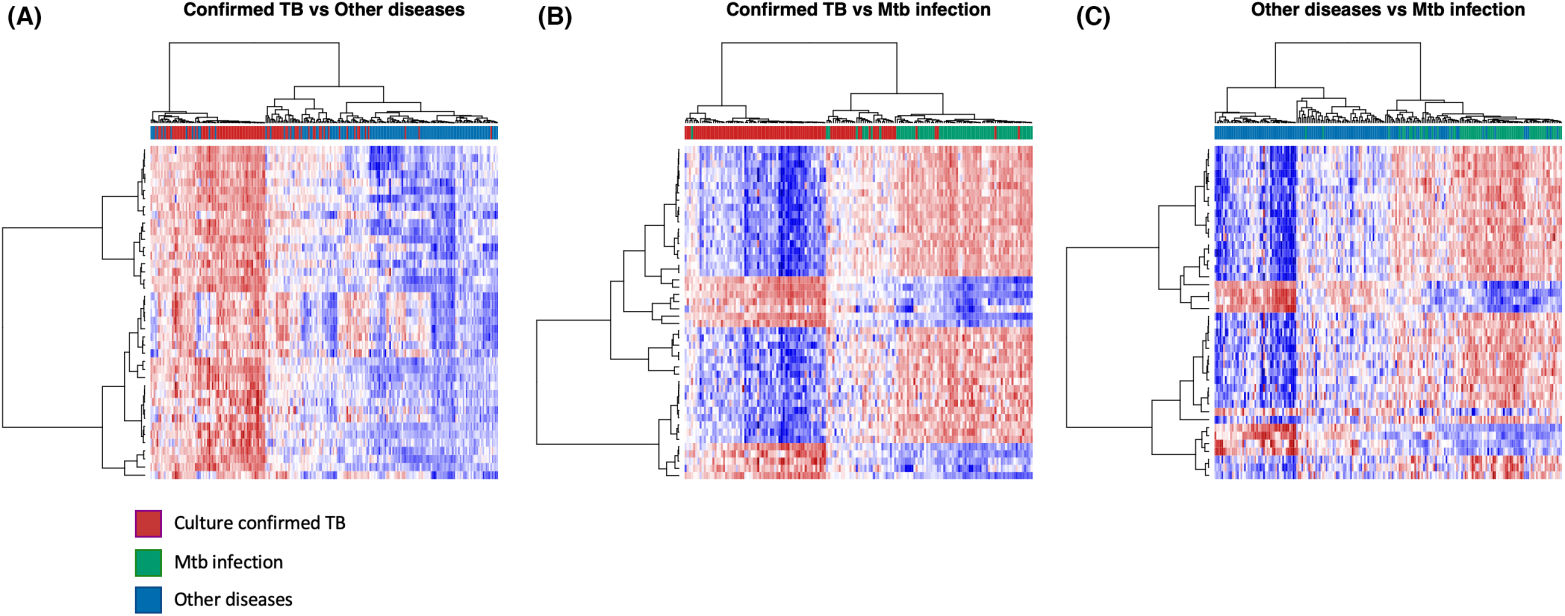
Heatmaps showing clustering (Unweighted Pair Group Method with Arithmetic Mean or UPGMA method) of the top 50 SDEs in (A) culture confirmed TB vs other diseases, (B) culture confirmed TB vs *Mtb* infection and (C) other diseases vs *Mtb* infection of the patients from South Africa, Malawi, and Kenya in Anderson et al datasets. Patients' clinical groups are highlighted at the bar on the top of each heatmap, with culture confirmed TB patients in red, patients with *Mtb* infection in green and patients with other diseases in blue. Only HIV uninfected patients have been included and shown on these heatmaps. Under‐expression is depicted in blue and over expression in red

**FIGURE 6:**
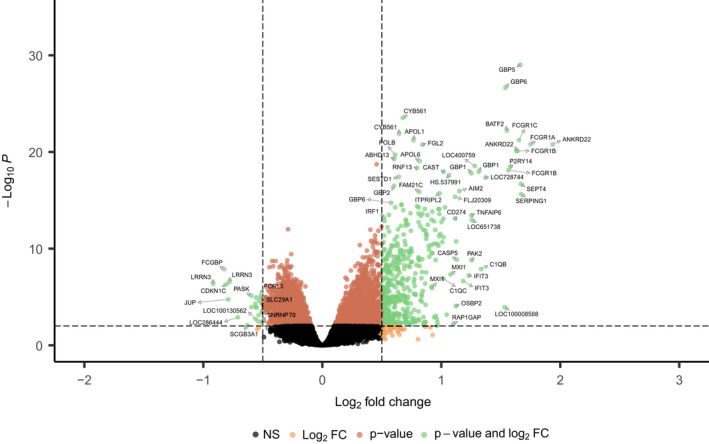
Volcano plot showing the significant genes identified comparing culture confirmed TB cases vs other diseases (OD) from Anderson et al South Africa, Malawi, and Kenya HIV uninfected patient datasets. Genes that passed the thresholds for absolute value of log2FoldChange >0.5 and adj‐p‐value <0.05, were colored in green

**FIGURE 7:**
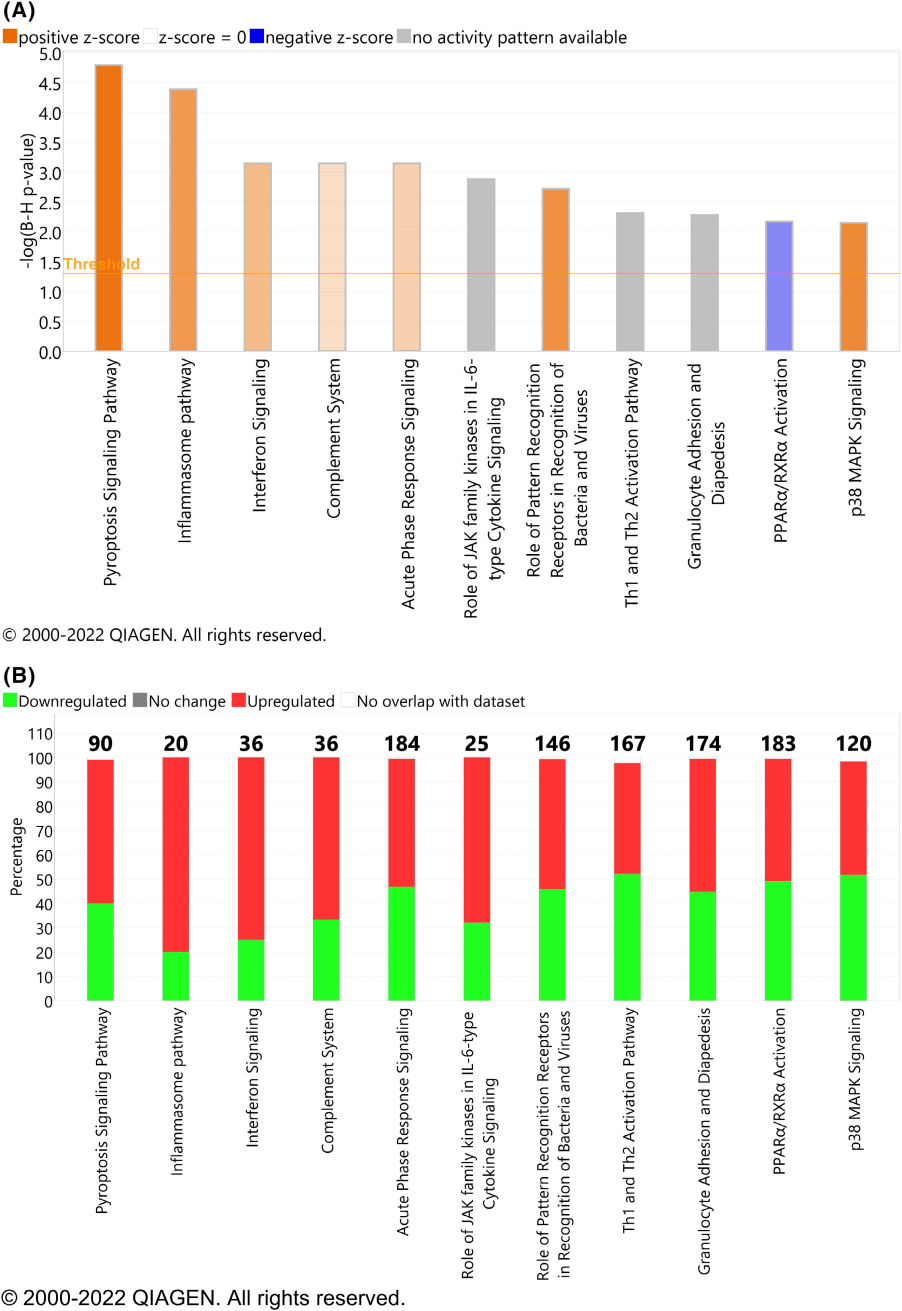
Canonical pathway analysis results comparing confirmed TB to other diseases (OD) using Anderson et al datasets. (A) The orange and blue‐colored bars in the bar chart indicate predicted pathway activation or predicted inhibition, respectively. Gray bars indicate pathways for which no prediction can be made due to insufficient evidence in the Knowledge Base for confident activity predictions across datasets. (B) Displays the number of molecules in the list of SDE genes, showing the up‐regulated (red), down‐regulated (green). The y‐axis represents the percentage of molecules that are present in a specific Canonical Pathway. The total number of molecules in the pathway is shown

**FIGURE 8:**
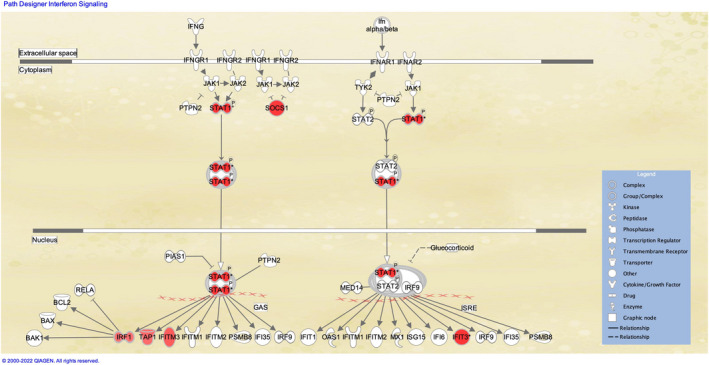
Canonical pathway example: Interferon Signaling Pathway. The molecules that are different shades of red color indicate up‐regulation in the comparison of TB vs other diseases

Parsimonious gene expression signatures are the product of feature selection algorithms, which try to maximize accuracy in segregation and simultaneously minimize the number of genes in the selected set based on specific criteria. Although they include key molecules that are differentially expressed between groups, they do not capture the full picture of perturbation in blood gene expression in different TB states. Here, we present the results of pathway analysis using Ingenuity Pathway Analysis (IPA) on SDE genes in TB vs. Other Diseases from the datasets presented in the Anderson and colleagues' study, as well as an overview of concordance/discordance of gene expression in TB and Other Diseases in relation to *Mtb* infection as the baseline. The analytical steps for quality control and pre‐processing were followed as reported in the original study, including background correction, variance stabilization and normalization, while the data from the HIV‐negative children from South Africa, Malawi and Kenya were combined.^118^, ^119^ Using a linear model corrected for age, gender and site, we identified 1021 transcripts that were SDE (adj. *P*‐value <0.0001, |log2FoldChange| > 0.25) between TB and other diseases (Figures 5 and 6). After performing ID mapping in IPA, 742 molecules (324 under‐expressed in TB and 418 over‐expressed in TB) were included in the pathway analysis. Figure 7 shows the most significant canonical pathways, with Benjamini‐Hochberg corrected *P*‐values, z‐scores indicating activation or inhibition. The significance indicates the probability of association of molecules from the list of SDE genes in the dataset with the canonical pathway by random chance alone. While Figure 8 demonstrates the interferon signaling pathway.

Differential expression analysis compares two patient groups in a direct way, identifying genes which are significantly different between the two in terms of expression. However, elements of the immune response as captured by the transcriptome may exhibit similarity or dissimilarity in comparison to a baseline (control) group. Here, we present the concordant and discordant genes in TB and other diseases in relation to *Mtb* infection as a baseline.^120^

**FIGURE 9:**
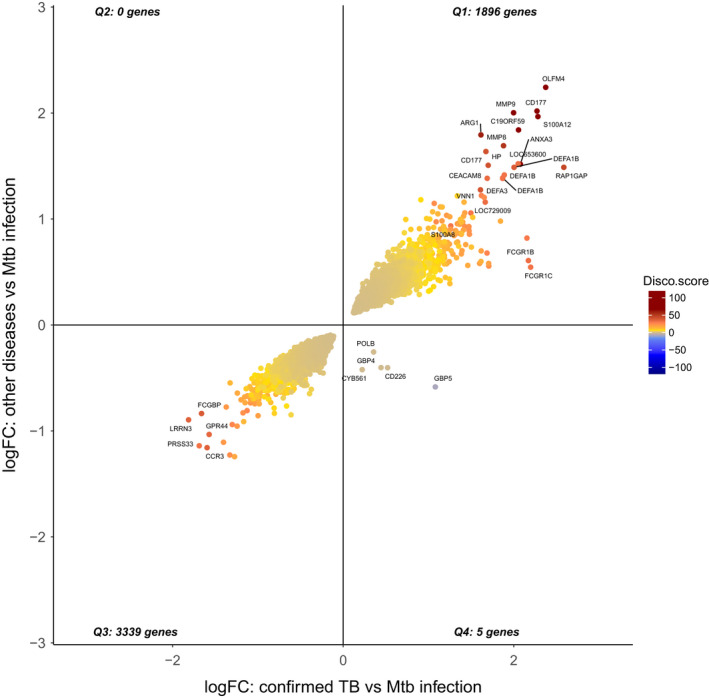
Concordance and discordance of the log2FoldChange of statistically differentially expressed genes in TB vs *Mtb* infection against the log2FoldChange of corresponding genes in OD vs *Mtb* infection from Anderson et al datasets. Each dot is colored according to the disco score and represents a gene: the stronger the red color the more concordantly regulated is the gene pair; the stronger the blue color, the more discordantly regulated is the gene pair

**FIGURE 10:**
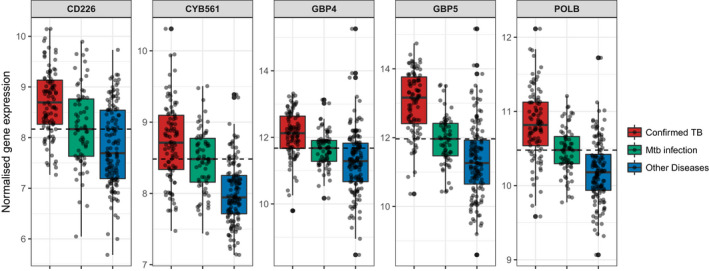
Boxplots of the 5 discordant genes between two comparisons TB vs *Mtb* infection (baseline) and OD vs *Mtb* infection (baseline) from Anderson et al datasets. The bar within each box indicates the median score, the bottom and top of the box indicate the interquartile range, the bars below and above the box are at a distance of 1.5 times the interquartile range from the upper and lower edges of the box, and the circles indicate outliers; the horizontal line across the graph indicates the median score of the *Mtb* infected group

**FIGURE 11:**
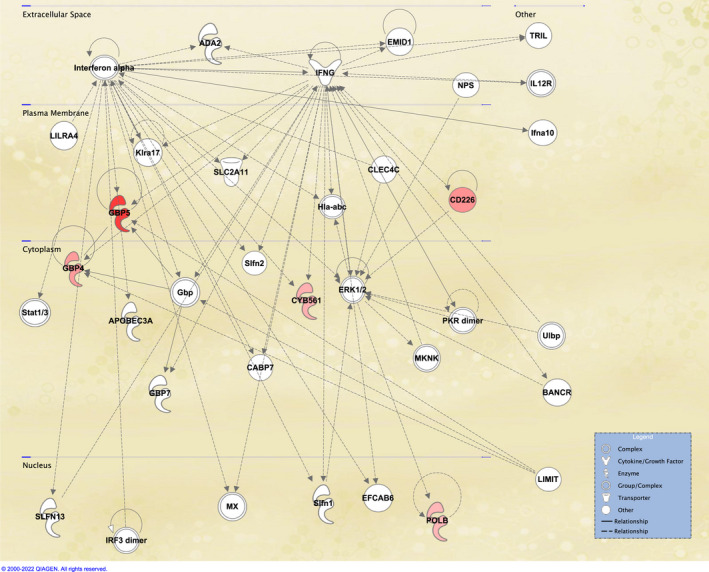
Molecular network, constructed from the discordant genes identified in the disco plot from Anderson et al dataset. The molecules that are different shades of red color indicate up‐regulation in the comparison of TB vs other diseases. The default number of molecules per network has been used, *n* = 35

The concordance‐discordance between the statistically differentially expressed genes (adjusted *P*‐value <0.01) between culture confirmed TB vs *Mtb* infection (11 533 SDE) and other diseases vs *Mtb* infection (5982 SDE) is shown in Figure 9, where a discordance and concordance score for homologous gene pairs was calculated based on their log2FoldChange and adjusted *P*‐value. The plot highlights the high degree of concordance of over‐ or under‐expression of SDE genes between TB disease and Other Diseases in relation to baseline. Remarkably, only 5 genes were found to be discordant (over‐expressed in TB vs. *Mtb* infection and under‐expressed in OD vs. *Mtb* infection), while 4 out these 5 genes have been previously proposed as TB‐specific transcriptomic biomarkers.^99^, ^110^ Normalized expression of these 5 genes is shown for TB disease, *Mtb* infection, and Other Diseases (Figure 10), while network analysis using Ingenuity Pathway Analysis indicated the 5 discordant genes form a network downstream of IFN‐a and IFN‐g (Figure 11).

### 6.2 Disseminated disease

Hemingway and colleagues employed blood gene expression profiling in children with TB meningitis (TBM) to identify temporal patterns of RNA during the course of the disease in comparison to healthy controls, and a second cohort of TBM and pulmonary TB (PTB) to explore differences in the host response in these two disease manifestations in children.^121^ The authors identified a decreased abundance of multiple genes in TBM patients in comparison with healthy controls, 68% of SDE genes were under‐expressed. The magnitude of differential abundance was less in PTB than in TBM. Most genes were involved in activation of leucocytes (*P =* 2.67E^−11^) and T‐cell receptor signaling (*P =* 6.56E^−07^). Multiple genes involved in T‐cell activation showed decreased abundance in children with TB, suggesting that childhood TB is associated with an acquired immune defect. Although in silico *deconvolution* revealed a reduction in CD4+ T lymphocytes in TBM, when compared with healthy controls, the differences in gene expression were not explained by the differing cell proportions. The suppression of genes in the T‐cell response pathway was associated with reduced T‐cell proliferative responses in vitro, indicating a functional impairment in responses is associated with the RNA transcriptional suppression.

In 2019, Rohlwink and colleagues conducted RNA‐Seq both on whole blood as well as brain and spinal CSF of children with TBM to identify patterns of differential expression against healthy controls and non‐TB cerebral infection controls in CSF.^122^ Differential gene expression analysis, functional canonical pathways and disease networks revealed that TBM is characterized by a significant increase in inflammasome activation, IL‐1 signaling and decrease in T‐cell activation in blood and neural injury in CSF. The findings corroborated the lack of an effective T‐cell response in pediatric TBM.

### 6.3 Transcriptomic profiles in children vs adolescents vs adults

The risk of progression from *Mtb* infection to disease is substantially lower in pre‐adolescent children above 4 years of age than in post‐pubescent adolescents and young adults.^123^ Blood gene expression profile analysis revealed that pre‐adolescent children had lower levels of myeloid‐associated pro‐inflammatory mediators than young adults. When compared with young adults, pre‐adolescent children had higher levels of IFN‐stimulated genes *IFNAR2*, *MX2*, *OAS1*, and *STAT2*, as well as B cells and M2 macrophages than adults.

To explore the difference between the immune response to *Mtb* between children and adults, Bah and colleagues analyzed 11 datasets comprising 1073 patients from Africa, Europe, and South America, including three datasets from children.^124^ Pathway analyses indicated that adults showed more IFN‐driven innate immune pathways and downregulated adaptive pathways. Childhood specific upregulated genes were associated with inflammasome IL1RN–IL1R2 suppression axis, glucose transport, CO2 and O2 release, and cell surface interaction pathways while downregulated genes were associated with mRNA translation, protein metabolism, and amino acid transport. In childhood TB, the immune inhibitory molecules, IL1RN and IL1R2 which inhibit functional IL1 signaling, and molecules involved in generation of an adaptive immune response (CD40LG, HAL‐DOB, CD28) that were downregulated is consistent with emerging evidence linking the cross talk between IL1 and type 1 IFN to TB and which provide potential targets for host directed therapy. This could explain the observation that children have less pronounced adaptive immune responses to *Mtb* compared with adults, potentially resulting in disseminated forms of the diseases seen more frequently in childhood TB.

## 7 MULTI‐OMICS APPROACHES

Despite the numerous insights obtained already by the analysis of host transcriptomic datasets, a single‐layer of ‐omics is unlikely to capture the complete biological complexity of disease.^125^ Integrating multiple‐omic levels, which represent multiple levels of biological organization, in a cross‐layer manner, enables a more accurate reconstruction of the dynamic molecular networks underpinning healthy and diseased states.^126^ The popularity of multi‐omic integration has grown rapidly during recent years, resulting in a vast range of integration methods for supervised and unsupervised analysis, using a range of statistical and machine learning approaches. By combining weak yet consistent alterations across different data layers, new insights have been obtained in cancer^127^ and COVID‐19 disease pathogenesis,^128^ while a recent study used three different ‐omic layers for biomarker identification for tuberculosis in the serum of adult patients with advanced HIV.^129^ Multi‐omic integrative studies could be key in furthering the understanding of pathogenesis and disease progression in childhood TB and can be used to provide combinations of host markers across different layers that could increase their discriminatory performance.

## 8 CLINICAL EVALUATION OF BIOMARKERS IN CHILDREN AND ADOLESCENTS

### 8.1 The challenge of unconfirmed TB

An important challenge for the development of biomarkers in childhood TB is the composition of children with unconfirmed TB. This is particularly relevant for the development of diagnostic tests for TB disease but has implications for prediction of future disease progression and treatment response on therapy for TB disease. Children with unconfirmed TB fulfil established consensus clinical and radiological criteria for disease but without microbiological confirmation.^17^, ^130^ Children with confirmed disease tend to have more severe disease than those with unconfirmed disease, the disease has usually been present for longer, symptoms are often more pronounced and there are likely more bacilli in the child. Many biomarker studies have been developed comparing children with confirmed disease to children felt very unlikely to have TB disease. When tests are evaluated in children with a diagnosis of unconfirmed TB, performance is generally less good. However, it is in these children that tests are most needed. There are several explanations for why performance may be less good, and it is likely that all are to some extent evident. First, all children with unconfirmed disease have TB but have a milder form of TB that has a less pronounced transcriptomic signature than children with confirmed disease. Second, some of the children with unconfirmed disease have TB but the overall signature for children with unconfirmed disease is “diluted” by the children who do not actually have TB at all. Finally, the interaction between host and pathogen in unconfirmed TB is actually subtly different to the interaction seen in confirmed disease.

One approach to try to statistically deal with the analysis of diagnostic studies in which the “gold” standard is imperfect is using latent class analysis.^131^ Schumacher and colleagues have previously employed Bayesian latent class analysis to estimate the diagnostic accuracy of mycobacterial culture, smear microscopy, Xpert MTB/RIF (Cepheid Inc.), TST, and chest radiography in childhood PTB, in a cohort of 748 hospitalized South African children. Transcriptomic signatures have not yet been evaluated using latent class analysis to define the reference standard. In the Anderson study,^99^ the authors addressed the challenge of evaluating the 51‐gene expression biomarker signature in children treated for TB, but without microbiological confirmation, by considering different scenarios where this population of children included varying combinations of “true” TB cases, and children incorrectly assigned as having TB by the clinical features. They used a range of estimates of the true prevalence of TB amongst those considered clinically as having highly probable TB, probable TB or possible TB, and used these estimates to evaluate the sensitivity of the TB Disease Risk Score, as well as to identify the children likely to have TB (High TB Disease Risk Score) and those unlikely to have TB (Low Disease Risk Score). The gradient in the performance of the risk score in the culture‐negative groups was consistent with the different degrees of diagnostic certainty in each group. Another approach would be to evaluate change in transcriptomic signature early in treatment to see if some children in the unconfirmed group have changes that cluster with the confirmed group and some have signatures that cluster with the unlikely TB group.

### 8.2 Development of point‐of‐care tests

The use of these biomarkers in a clinical decision process either as standalone diagnostic tools or in conjunction with other tools needs to be studied further. Ultimately, prospective studies would be required in which the decisions about whether and when to initiate TB treatment are evaluated when using the new biomarkers. A concern in using transcriptional signatures as clinical diagnostic tools in resource poor settings is the complexity, cost, and time needed for the current methodologies for isolating and quantifying RNA from blood. The approaches described above have generally collected samples from children/adolescents and then divided the population into distinct clinical groups before identifying the minimal number of transcripts that can effectively discriminate these groups. A discovery/test approach is commonly used prior to validation in an external cohort. However, the next step necessary to make a signature like this useful clinically is to translate it into a true point‐of‐care test (POCT). For RT‐qPCR based platforms, individual primers to each transcript need to be designed and these need to be then tested experimentally and validated externally. Platforms need to be robust, affordable, reliable, and able to be used in low resource settings with intermittent power supply and frequently at the extremes of temperature and humidity. Only a relatively small number of transcripts (typically fewer than ten) can be included in RT‐qPCR POCTs for feasibility of engineering. POCTs have mainly, but not exclusively, been developed by commercial groups, with expertise in diagnostic test development and steps are currently being made toward cost‐effective ways of measuring gene expression in the field efficiently by using recent advances in biotechnology and engineering.^132^ An early‐prototype cartridge‐based assay, the “Xpert MTB Host Response” Cepheid (Sunnyvale, CA, USA) measuring the Sweeney 3‐gene signature discriminated adults with TB disease from controls amongst people living with HIV (>18 years) with an AUC of 0.89 (CI_95%_ 0.83–0.94) and AUC of 0.84 (CI_95%_ 0.79–0.89) for active case finding in a high burden setting.^133^, ^134^ Similar studies on pediatric populations are needed to assess the potential of these signatures in pediatric TB.

### 8.3 Evaluation in real‐time as point‐of‐care tests

Once a POCT has been developed it must be evaluated clinically to determine discriminatory accuracy in real‐world conditions, ease‐of‐use, resource utilization, time to clinical decision‐making and ultimately clinical outcome. Direct translation into clinical studies in which treatment decisions have been led by biomarker‐based POCTs can guide preventive therapy in healthy individuals. The CORTIS trial recruited 2923 HIV‐negative adults. Individuals with a positive 11‐transcript PT‐qPCR test (the RISK11 signature) were randomized to TPT or no treatment while a sub‐set of individuals with a negative RISK11 signature were followed with no treatment.^111^ Those with a positive RISK11 signature were at increased risk of having prevalent TB and also of progressing to TB in the subsequent 15 months. However, when comparing rates of incident TB between the RISK11‐positive adults treated with TPT and those not treated, the risk of disease was not reduced. The research team are continuing to explore reasons for this unexpected finding.

Unlike in adults, however, it can be challenging to conduct a study in children where clinical decisions, particularly around which symptomatic child should be treated for TB disease, are based on a transcriptomic POCT. In addition, any test that is to be used to guide clinical decision‐making would need to be approved by medical regulators in the country used. A potential interim step would be to carry out a Simulated Clinical Impact study in which the biomarker POCT is conducted in real‐time in parallel to the routine standard of care decision‐making. In that way, real patient information (from children recruited to a prospective cohort) is later presented to independent clinicians, and their clinical decisions, use of investigations, and therapeutic choices are compared when POCT results are made available at certain timepoints post presentation of child. Ultimately, biomarker tests need to be evaluated in comparison to other diagnostic approaches with the hard clinical endpoints being the outcomes of interest. Additional data on time to treatment initiation, preferences of children, parents, and healthcare worker, and costs need also to considered.

### 8.4 Integration into treatment decision algorithms

**FIGURE 12:**
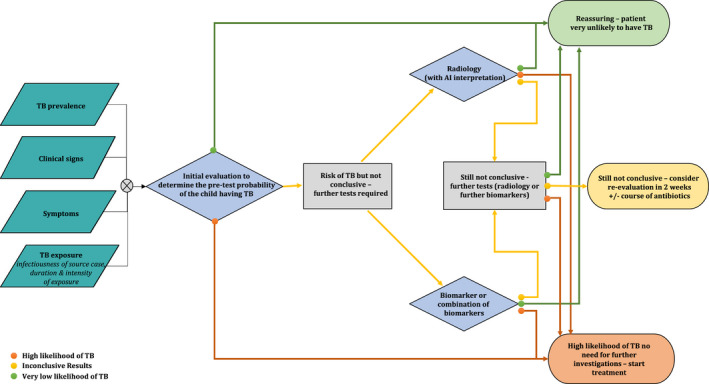
Illustration of an integrated TB treatment decision algorithm including biomarkers

In most clinical decision‐making, the pre‐test probability of a disease is combined with a test result to arrive at a post‐test probability that then informs whether treatment should be started. For many children with presumptive TB, information from the clinical history and examination alone is sufficient to either reassure the healthcare worker that the child does not have TB or that the child is highly likely to have TB and should be started on treatment even if further tests were negative. In these instances, it is not necessary or helpful to perform testing. If the healthcare worker is not sufficiently reassured or convinced of the need to start treatment, then biomarker testing can occur, but the interpretation of that test result should include data from the clinical history and presentation as pre‐test probability of disease. In addition, if further tests are performed after the first biomarker (which might include other biomarkers or radiological investigations) the interpretation of these results should include clinical information as well as results from any tests already performed. So, in addition to evaluating biomarkers for their ability to distinguish clinical states in children, it is important going forward to evaluate how biomarkers can be incorporated into treatment‐decision algorithms. Data driven approaches have been developed to combine clinical, radiological, and microbiological data for the evaluation of child TB.^135^, ^136^ To date, these have not included transcriptomic biomarkers. An illustrative example is shown in Figure 12.

## 9 CONCLUSIONS

Over the last ten years, transcriptomic approaches have led to the generation of multiple biomarkers in adults that can predict future TB disease progression, discriminate TB from other disease and monitor treatment response. Transcriptomics has also allowed for novel insights into the pathogenesis of *Mtb* infection and disease as well as better understanding of the host response to this pathogen. Work in children and adolescents has lagged but several seminal studies have demonstrated that the host response to *Mtb* varies with age and the discovery of child‐specific biomarkers requires child‐specific studies. As these signatures are developed, they will need to be translated first into POCTs and then rigorously evaluated in the relevant clinical contexts, alone, and as part of integrated algorithms. In addition to the discovery of pediatric and adolescent biomarkers, transcriptomic studies in children are beginning to help us understand the biology of *Mtb* infection and disease in this age group, which will be vital to develop better vaccines and therapeutics. Transcriptomics has the potential to substantially contribute to meeting global End TB targets.

## CONFLICTS OF INTEREST

MK, ML, and TS hold patents on several gene expression signatures. CB, OV, and JAS have no conflicts of interest.

## Data Availability

Data sharing is not applicable to this article as no new data were created or analyzed in this study. Data presented in Figures 5, 6, 7, 8, 9 are publically available in Anderson et al [99].
